# Self-Reported Hypertension and Use of Antihypertensive Medication Among Adults — United States, 2005–2009

**Published:** 2013-04-05

**Authors:** Jing Fang, Carma Ayala, Fleetwood Loustalot, Shifan Dai

**Affiliations:** Div for Heart Disease and Stroke Prevention, National Center for Chronic Disease Prevention and Health Promotion, CDC

Hypertension affects one third of adults in the United States (1) and is a major risk factor for heart disease and stroke (2). A previous report found differences in the prevalence of hypertension among racial/ethnic populations in the United States; blacks had a higher prevalence of hypertension, and Hispanics had the lowest use of antihypertensive medication (3). Recent variations in geographic differences in hypertension prevalence in the United States are less well known (4). To assess state-level trends in self-reported hypertension and treatment among U.S. adults, CDC analyzed 2005–2009 data from the Behavioral Risk Factor Surveillance System (BRFSS). The results indicated wide variation among states in the prevalence of self-reported diagnosed hypertension and use of antihypertensive medications. In 2009, the age-adjusted prevalence of self-reported hypertension ranged from 20.9% in Minnesota to 35.9% in Mississippi. The proportion reporting use of antihypertensive medications among those who reported hypertension ranged from 52.3% in California to 74.1% in Tennessee. From 2005 to 2009, nearly all states had an increased prevalence of self-reported hypertension, with percentage-point increases ranging from 0.2 for Virginia (from 26.9% to 27.1%) to 7.0 for Kentucky (from 27.5% to 34.5%). Overall, from 2005 to 2009, the prevalence of self-reported hypertension among U.S. adults increased from 25.8% to 28.3%. Among those reporting hypertension, the proportion using antihypertensive medications increased from 61.1% to 62.6%. Increased knowledge of the differences in self-reported prevalence of hypertension and use of antihypertensive medications by state can help in guiding programs to prevent heart disease, stroke, and other complications of uncontrolled hypertension, including those conducted by state and local public health agencies and health-care providers.

BRFSS is a state-based telephone survey of health behaviors among adults aged ≥18 years.* The survey has been conducted by state health departments, with assistance from CDC, since 1984. Questions on hypertension are asked in odd-numbered years. Since 2005, two questions about hypertension have been included in BRFSS. The first question is, “Have you ever been told by a doctor, nurse, or other health professional that you have high blood pressure?” Respondents who answer “yes” to the first question are then asked, “Are you currently taking medicine for your high blood pressure?” These questions were used to assess prevalence of self-reported hypertension and proportion reporting antihypertensive medication use among those with reported hypertension in 2005, 2007, and 2009. Estimates were calculated for the United States overall and for the 50 states and the District of Columbia. In addition to analysis by state, estimates were analyzed by age group, sex, race/ethnicity,† and level of education. Age-adjusted estimates were calculated using the 2000 U.S. standard population. Linear trends were assessed using orthogonal polynomial coefficients, and results were considered significant at p<0.05.

Median state response rates for BRFSS were 51.1% (range: 34.6%–67.4%) in 2005, 50.6% (range: 26.9%–65.4%) in 2007, and 52.5% (range: 37.9%–66.9%) in 2009. Total respondents were 356,112 in 2005, 430,912 in 2007, and 432,617 in 2009. State sample sizes ranged from 2,432 in 2009 (Alaska) to 39,549 in 2007 (Florida).

From 2005 to 2009, overall age-adjusted prevalence of self-reported hypertension in the United States increased from 25.8% to 28.3% (Table 1). Self-reported hypertension ranged from 21.1% (Colorado) to 33.5% (Mississippi) in 2005, and from 20.9% (Minnesota) to 35.9% (Mississippi) in 2009. From 2005 to 2009, nearly all states had an increased prevalence of self-reported hypertension, with percentage-point increases ranging from 0.2 for Virginia (from 26.9% to 27.1%) to 7.0 for Kentucky (from 27.5% to 34.5%). In 2009, the prevalence of self-reported hypertension was, in general, higher in southern states and lower in western states (Figure).

Among those with self-reported hypertension, the estimated number of participants reporting use of antihypertensive medications was 45,023,301 in 2005, 50,191,337 in 2007, and 53,602,447 in 2009; the proportion increased from 61.1% (2005) to 62.6% (2009). In 2009, among those with self-reported hypertension, the proportion reporting current use of antihypertensive medication was highest in Tennessee (74.1%) and lowest in California (52.3%); however, Tennessee showed no significant change in reported antihypertensive medication use from 2005 to 2009, whereas California had a significant increase, from 48.0% to 52.3%. As with self-reported hypertension, the proportion of participants reporting use of antihypertensive medication generally was higher in southern states and lower in western states (Figure). States that showed significant increases in use of antihypertensive medications included California, Iowa, and Michigan, whereas Kentucky, Nebraska, and Rhode Island had significant decreases.

By selected characteristics, self-reported hypertension prevalence in 2009 was significantly higher among persons aged ≥65 years (59.6%) compared with persons aged 18–44 years (13.3%) and 45–64 years (37.1%); among men (30.3%) compared with women (26.2%); among blacks (39.6%) compared with American Indian/Alaska Natives (32.0%), Hispanics (27.6%), whites (27.1%), and Asian/Pacific Islanders (24.0%); and among those with less than a high school education (33.6%) compared with those with a high school education (31.4%), those with some college (29.2%), and those with a college degree or higher (23.8%). From 2005 to 2009, the prevalence of self-reported hypertension increased for all sociodemographic subgroups, although the linear trends were not significant for Hispanics, Asian/Pacific Islanders, and American Indian/Alaska Natives (Table 1).

Among persons reporting hypertension in 2009, the proportion reporting antihypertensive medication use was significantly higher among persons aged ≥65 years (94.1%) compared with those aged 18–44 years (45.1%) and 45–64 years (82.3%); among women (66.9%) compared with men (59.9%); and among blacks (71.6%) compared with Hispanics (55.2%) (Table 2). From 2005 to 2009, significant increases in self-reported use of antihypertensive medication among those reporting hypertension were observed among blacks (from 67.0% to 71.6%) and Hispanics (from 51.2% to 55.2%).

## Editorial Note

The findings in this report, using BRFSS data, indicate that from 2005 to 2009, a small but significant increase in the prevalence of self-reported hypertension was observed among U.S. adults. Among those with self-reported hypertension, the proportion who reported use of antihypertensive medication also increased significantly.

In 2011, a report based on results from the National Health and Nutrition Examination Survey (NHANES) showed that among adults aged ≥18 years, the prevalence of measured hypertension did not increase significantly from 1999–2002 to 2005–2008; however, the use of antihypertensive medication and control of hypertension showed significant increases (1). The prevalence of measured hypertension in NHANES did not increase during 1999–2008 (1); therefore, the increase in self-reported hypertension described in the current report likely is related to an increase in the awareness of hypertension. Measured blood pressure is not available with BRFSS surveys; therefore, hypertension control could not be assessed in the current report. The findings in this report show that among persons with hypertension, the proportion reporting antihypertensive medication use increased overall from 2005 to 2009; however, only a few states showed significant increases or decreases in the proportion reporting antihypertensive medication use.

What is already known on this topic?

Hypertension is a major risk factor for cardiovascular disease. In the United States, hypertension affects approximately one third of the adult population. Differences in prevalence of hypertension and use of antihypertensive medications exist among states and sociodemographic subgroups. As with this report, U.S. states and territories frequently use Behavioral Risk Factor Surveillance System data to aid in tracking priority health conditions and behaviors and to support the targeting of limited programmatic resources to high-prevalence areas.

What is added by this report?

From 2005 to 2009, the prevalence of self-reported hypertension among U.S. adults increased from 25.8% to 28.3%. Among those with self-reported hypertension, use of antihypertensive medications increased from 61.1% to 62.6%. Among states, rates of self-reported hypertension in 2009 ranged from 20.9% to 35.9%.

What are the implications for public health practice?

Improving hypertension awareness and initiating appropriate treatment are important to increase blood pressure control and reduce risk for heart disease and stroke. The findings in this study provide public health practitioners information to help target blood pressure control efforts. Public health officials, particularly in those states with a high prevalence of hypertension, should consider a coordinated and multifactorial approach to blood pressure control with focused attention in areas including sodium reduction, health systems strategies such as promotion of the collection and use of quality measures, promotion of team-based care, and community-clinical linkages.

Substantial differences among states were observed for self-reported hypertension prevalence, in general, the prevalence was higher in southern states than in other regions. Use of antihypertensive medication varied by state, but overall BRFSS estimates generally were consistent with other national estimates (5–7). The recent REasons for Geographic and Racial Differences in Stroke (REGARDS) study found that, compared with whites, black participants were more aware of hypertension and more likely to be treated. However, among those treated, blacks were less likely than whites to have their blood pressure controlled (5). The high prevalence of hypertension in the southern states found in this study is in the “stroke belt,” a geographically identified region of high stroke morbidity and mortality, and likely is contributing to the disparate burden of disease in the region (8). The findings by sex were similar to results from NHANES 2005–2008, which found that anti-hypertensive treatment was lower among men than women (7).

The findings in this report are subject to at least three limitations. First, data were self-reported, and hypertension and use of antihypertensive medications were not verified independently. Second, BRFSS surveys only noninstitutionalized persons with landline telephones; in 2009, 24.5% of U.S. households only had cellular telephone service (9). Finally, median state response rates for BRFSS were low; however, BRFSS provides the only available state-specific estimates of hypertension prevalence and antihypertensive medication use.

Hypertension is a major modifiable risk factor for cardiovascular disease, and improving awareness of hypertension is an important first step to treating and controlling hypertension and preventing heart disease and stroke. Clinical guidelines for hypertension management emphasize the control of hypertension through participation in healthy lifestyle behaviors, and using appropriate and specific antihypertensives medications with integrated clinical systems to support sustained adherence (2). A CDC goal is to increase public health interventions in clinical and community settings to reduce the deleterious effects of hypertension by increasing awareness and control of high blood pressure.§ One effective intervention is the Community Preventive Services Task Force recommendation for use of team-based care to improve blood pressure control.¶ Currently, 41 states receive CDC funding to develop and implement heart disease and stroke prevention programs.** CDC’s National Heart Disease and Stroke Prevention Program works to increase prevention and control of high blood pressure through sodium reduction, health system strategies such as collection and use of quality measures, promotion of team-based care, and community-clinical linkages.

In addition, the Million Hearts initiative, a public and private partnership co-led by CDC and the Centers for Medicare and Medicare Services, targets blood pressure control and seeks to align and coordinate resources across community and clinical settings (10). Increasing awareness of hypertension, improving hypertension control, and encouraging adherence to evidence-based practices addressing hypertension are needed, especially in those states with higher prevalence of hypertension and lower proportion of use of antihypertensive medications.

## Figures and Tables

**FIGURE:**
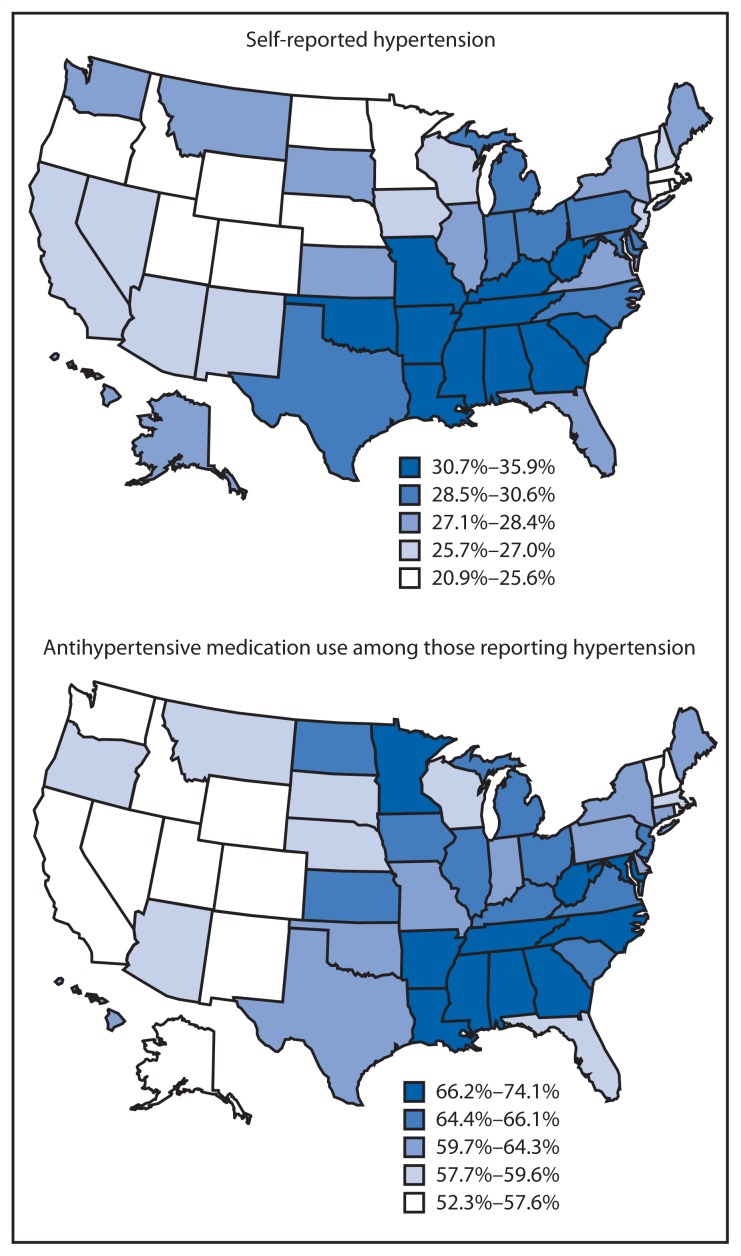
Age-adjusted prevalence of self-reported hypertension among adults and the proportion of those participants reporting use of antihypertensive medication, by state — Behavioral Risk Factor Surveillance System, United States, 2009

**TABLE 1:** Age-adjusted prevalence of self-reported hypertension among adults, by sociodemographic characteristics and location — Behavioral Risk Factor Surveillance System, United States, 2005–2009

Characteristic/Location	2005		2007		2009		Percentage- point change 2005 to 2009	% change 2005 to 2009	p-value for trend
		
	%	(95% CI)	%	(95% CI)	%	(95% CI)			
**Total**	**25.8**	**(25.6–26.1)**	**26.9**	**(26.7–27.2)**	**28.3**	**(28.0–28.5)**	**2.5**	**9.7**	**0.001**
**Age group (yrs)**									
18–44	10.8	(10.5–11.2)	11.8	(11.5–12.2)	13.3	(12.9–13.7)	2.5	23.1	<0.001
45–64	35.0	(34.5–35.5)	36.2	(35.8–36.7)	37.1	(36.7–37.5)	2.1	6.0	<0.001
≥65	56.0	(55.4–56.7)	58.1	(57.6–58.7)	59.6	(59.2–60.1)	3.6	6.4	<0.001
**Sex**									
Men	26.8	(26.4–27.2)	28.5	(28.1–28.9)	30.3	(29.9–30.7)	3.5	13.1	<0.001
Women	24.7	(24.4–25.0)	25.3	(25.1–25.6)	26.2	(25.9–26.5)	3.5	6.1	<0.001
**Race/Ethnicity** *									
White	24.6	(24.3–24.8)	25.8	(25.6–26.0)	27.1	(26.8–27.3)	2.5	10.2	<0.001
Black	36.3	(35.4–37.3)	38.1	(37.2–39.0)	39.6	(38.7–40.6)	3.3	9.1	<0.001
Asian/Pacific Islander	21.3	(19.0–23.8)	21.5	(19.4–23.8)	24.0	(22.4–25.7)	2.7	12.7	0.066
American Indian/Alaska Native	30.8	(28.1–33.8)	31.0	(28.6–33.4)	32.0	(29.8–34.3)	1.2	3.9	0.536
Hispanic	26.4	(25.3–27.5)	26.4	(25.4–27.4)	27.6	(26.8–28.5)	1.2	4.5	0.092
**Education**									
<High school	31.2	(30.2–32.2)	30.6	(29.6–31.5)	33.6	(32.7–34.6)	2.4	7.7	<0.001
High school	28.1	(27.7–28.6)	30.1	(29.6–30.6)	31.4	(30.9–31.9)	3.3	11.7	<0.001
Some college	26.2	(25.7–26.7)	27.8	(27.3–28.3)	29.2	(28.8–29.7)	3.0	11.5	<0.001
≥College	21.5	(21.1–21.9)	22.5	(22.1–22.9)	23.8	(23.4–24.2)	2.3	10.7	<0.001
**State/Area**									
Alabama	30.2	(28.6–31.9)	31.9	(30.5–33.3)	34.0	(32.4–35.6)	3.8	12.4	0.001
Alaska	23.6	(21.5–25.7)	27.1	(24.7–29.7)	27.9	(25.7–30.0)	4.3	18.5	0.006
Arizona	22.1	(20.4–24.0)	24.2	(22.3–26.3)	25.7	(23.8–27.6)	3.6	16.0	0.007
Arkansas	27.9	(26.7–29.2)	29.8	(28.5–31.2)	32.2	(30.4–34.1)	4.3	15.3	<0.001
California	26.5	(25.2–27.9)	25.8	(24.5–27.1)	26.1	(25.3–26.9)	−0.4	−1.7	0.569
Colorado	21.1	(20.1–22.1)	22.0	(21.3–22.8)	22.7	(21.8–23.6)	1.6	7.6	0.019
Connecticut	22.4	(21.2–23.7)	24.5	(23.3–25.8)	25.4	(24.0–26.8)	3.0	13.3	0.002
Delaware	27.3	(25.7–28.9)	28.2	(26.6–29.8)	29.1	(27.4–30.8)	1.8	6.5	0.135
District of Columbia	28.5	(26.9–30.2)	29.1	(27.5–30.8)	27.0	(25.5–28.5)	−1.5	−5.4	0.184
Florida	25.0	(23.7–26.2)	25.2	(24.3–26.1)	27.7	(26.3–29.2)	2.7	11.0	0.004
Georgia	28.1	(26.8–29.4)	31.0	(29.7–32.2)	31.6	(29.8–33.4)	3.5	12.4	0.003
Hawaii	23.2	(21.8–24.5)	27.2	(25.9–28.6)	28.4	(27.1–29.8)	5.2	22.9	<0.001
Idaho	23.7	(22.5–24.9)	25.9	(24.5–27.2)	25.4	(24.1–26.8)	1.7	7.2	0.065
Illinois	25.4	(24.2–26.7)	27.6	(26.2–28.9)	28.4	(27.0–29.8)	3.0	11.7	0.002
Indiana	25.7	(24.5–26.9)	27.0	(25.8–28.3)	30.3	(29.2–31.4)	4.6	17.6	<0.001
Iowa	22.9	(21.7–24.0)	25.0	(23.8–26.2)	26.1	(24.8–27.5)	3.2	14.3	<0.001
Kansas	23.7	(22.8–24.6)	26.1	(25.1–27.1)	27.6	(26.8–28.3)	3.9	16.4	<0.001
Kentucky	27.5	(26.2–28.9)	28.6	(27.2–30.0)	34.5	(33.0–36.1)	7.0	25.5	<0.001
Louisiana	29.3	(27.6–31.0)	31.3	(30.0–32.7)	34.6	(33.3–35.9)	5.3	18.2	<0.001
Maine	24.0	(22.6–25.4)	26.5	(25.1–27.8)	27.3	(26.1–28.5)	3.3	14.0	<0.001
Maryland	25.7	(24.6–26.7)	28.4	(27.2–29.6)	28.6	(27.3–29.8)	2.9	11.3	<0.001
Massachusetts	24.1	(23.0–25.2)	25.1	(24.3–25.8)	24.5	(23.6–25.5)	0.4	1.7	0.57
Michigan	27.1	(26.3–28.0)	27.8	(26.6–29.0)	28.7	(27.6–29.8)	1.6	5.7	0.03
Minnesota	21.8	(20.4–23.3)	21.0	(19.9–22.2)	20.9	(19.7–22.2)	−0.9	−4.2	0.346
Mississippi	33.5	(32.0–34.9)	33.3	(32.1–34.5)	35.9	(34.7–37.0)	2.4	7.1	0.013
Missouri	26.4	(24.9–28.0)	28.2	(26.6–29.9)	28.9	(27.3–30.5)	2.5	9.3	0.032
Montana	22.5	(21.1–23.9)	23.4	(22.2–24.6)	25.7	(24.5–27.0)	3.2	14.6	<0.001
Nebraska	23.8	(22.8–24.9)	25.4	(24.0–26.8)	25.5	(24.4–26.6)	1.7	7.1	0.027
Nevada	24.2	(22.2–26.2)	26.9	(25.2–28.8)	26.6	(24.6–28.6)	2.4	9.9	0.099
New Hampshire	22.5	(21.4–23.6)	24.6	(23.5–25.8)	26.9	(25.4–28.4)	4.4	19.7	<0.001
New Jersey	24.3	(23.5–25.2)	26.7	(25.3–28.2)	26.7	(25.6–27.8)	2.4	9.9	<0.001
New Mexico	22.3	(21.2–23.5)	24.8	(23.5–26.1)	25.8	(24.6–27.0)	3.5	15.6	<0.001
New York	24.9	(23.9–26.0)	26.2	(25.0–26.2)	27.5	(26.1–28.9)	2.6	10.3	0.004
North Carolina	29.1	(28.3–29.9)	28.4	(27.5–29.2)	30.6	(29.5–31.8)	1.5	5.3	0.03
North Dakota	21.8	(20.6–23.0)	24.5	(23.2–25.7)	25.3	(24.0–26.6)	3.5	15.8	<0.001
Ohio	25.9	(24.5–27.3)	26.9	(25.9–27.9)	29.8	(28.6–31.1)	3.9	15.2	<0.001
Oklahoma	29.0	(27.9–30.1)	29.9	(28.7–31.1)	32.2	(31.1–33.5)	3.3	11.3	<0.001
Oregon	22.9	(22.2–23.7)	25.4	(24.1–26.8)	25.6	(24.1–27.2)	2.7	11.7	0.002
Pennsylvania	25.1	(24.1–26.1)	25.7	(24.6–26.9)	29.2	(28.0–30.5)	4.1	16.4	<0.001
Rhode Island	25.5	(24.0–27.0)	27.1	(25.7–28.5)	28.7	(27.3–30.1)	3.2	12.8	0.002
South Carolina	30.8	(29.8–31.8)	29.3	(28.2–30.4)	31.1	(29.6–32.6)	0.3	0.9	0.762
South Dakota	23.9	(22.9–25.0)	24.1	(23.0–25.2)	27.8	(26.5–29.2)	3.9	16.3	<0.001
Tennessee	29.6	(27.9–31.3)	32.0	(30.2–33.8)	30.8	(29.0–32.7)	1.2	4.3	0.316
Texas	25.6	(24.4–26.7)	28.3	(27.4–29.2)	29.6	(28.4–30.9)	4.0	16.0	<0.001
Utah	21.2	(20.0–22.4)	22.4	(21.2–23.7)	25.5	(24.5–26.5)	4.3	20.1	<0.001
Vermont	22.7	(21.8–23.7)	23.3	(22.2–24.5)	25.1	(23.9–26.3)	2.4	10.4	0.003
Virginia	26.9	(25.5–28.4)	26.5	(25.1–28.0)	27.1	(25.5–28.8)	0.2	0.7	0.867
Washington	24.1	(23.5–24.8)	25.2	(24.6–25.8)	27.5	(26.7–28.2)	3.4	13.8	<0.001
West Virginia	28.8	(27.3–30.3)	30.4	(28.9–31.9)	34.6	(33.1–36.3)	5.8	20.2	<0.001
Wisconsin	24.3	(23.1–25.6)	25.2	(23.9–26.5)	26.4	(24.7–28.1)	2.1	8.5	0.054
Wyoming	22.6	(21.5–23.8)	24.1	(22.9–25.2)	25.0	(23.9–26.2)	2.4	10.7	0.004

**Abbreviation:** CI = confidence interval.

* In this report, persons identified as Hispanic might be of any race. Persons identified as black, white, Asian/Pacific Islander, or American Indian/Alaska Native are non-Hispanic. The five racial/ethnic categories are mutually exclusive.

**TABLE 2:** Among participants with self-reported hypertension, age-adjusted proportion of those reporting use of antihypertensive medication among adults, by sociodemographic characteristics and location — Behavioral Risk Factor Surveillance System, United States, 2005–2009

Characteristic/Location	2005		2007		2009		Percentage-point change 2005 to 2009	% change 2005 to 2009	p-value for trend
		
	%	(95% CI)	%	(95% CI)	%	(95% CI)			
**Total**	**61.1**	**(60.3–61.9)**	**63.2**	**(62.4–64.0)**	**62.6**	**(61.8–63.5)**	**1.5**	**2.5**	**0.016**
**Age group (yrs)**									
18–44	43.6	(42.1–45.1)	47.5	(45.9–49.1)	45.1	(43.6–46.6)	1.5	3.4	0.172
45–64	80.0	(79.2–80.8)	82.2	(81.5–82.8)	82.3	(81.7–82.8)	2.3	2.9	<0.001
≥65	93.0	(92.4–93.4)	93.9	(93.6–94.3)	94.1	(93.8–94.3)	1.1	1.2	<0.001
**Sex**									
Men	58.0	(56.8–59.1)	61.1	(59.9–62.2)	59.9	(58.8–61.1)	1.9	3.3	0.014
Women	65.2	(64.0–66.4)	66.0	(64.9–67.1)	66.9	(65.7–68.0)	1.7	2.6	0.054
**Race/Ethnicity** *									
White	62.4	(61.4–63.4)	64.3	(63.3–65.2)	62.4	(61.5–63.3)	0.0	0.0	0.964
Black	67.0	(65.1–68.0)	69.5	(67.4–71.4)	71.6	(69.0–74.3)	4.6	6.9	0.004
Asian/Pacific Islander	61.4	(55.5–67.0)	60.1	(54.1–65.8)	60.2	(55.1–65.0)	−1.2	−2.0	0.752
American Indian/Alaska Native	59.8	(52.4–66.8)	61.9	(56.5–67.0)	61.8	(56.3–67.1)	2.0	3.3	0.668
Hispanic	51.2	(48.6–53.7)	54.9	(52.5–57.3)	55.2	(53.0–57.3)	4.0	7.8	0.019
**Education**									
<High school	56.7	(54.3–59.2)	57.6	(55.2–60.1)	59.6	(57.1–62.2)	2.9	5.1	0.106
High school	62.4	(60.9–63.8)	63.5	(62.0–64.9)	62.9	(61.3–64.4)	0.5	0.8	0.645
Some college	61.3	(59.9–62.7)	64.0	(62.6–65.4)	62.8	(61.4–64.1)	1.5	2.4	0.138
≥College	61.6	(59.8–63.3)	64.7	(62.7–66.6)	62.6	(61.1–64.1)	1.0	1.6	0.373
**State/Area**									
Alabama	68.9	(63.6–73.8)	78.7	(73.7–83.0)	72.5	(67.2–77.3)	3.6	5.2	0.325
Alaska	54.3	(49.3–59.2)	59.4	(52.8–65.7)	53.8	(47.7–59.8)	−0.5	−0.9	0.907
Arizona	60.8	(52.7–68.3)	59.1	(51.4–66.4)	58.9	(52.7–64.9)	−2.0	−3.1	0.712
Arkansas	65.6	(61.7–69.3)	70.2	(65.1–74.9)	67.6	(61.1–73.5)	2.0	3.1	0.59
California	48.0	(45.0–51.1)	52.3	(48.4–56.2)	52.3	(49.9–54.6)	4.3	8.8	0.032
Colorado	55.5	(51.6–59.4)	57.0	(53.7–60.2)	57.0	(53.6–60.3)	1.5	2.6	0.588
Connecticut	64.8	(58.2–70.9)	64.9	(59.8–69.7)	59.9	(55.5–64.1)	−4.9	−7.7	0.205
Delaware	66.2	(61.8–70.4)	62.5	(58.1–66.8)	62.7	(58.2–67.0)	−3.5	−5.4	0.261
District of Columbia	61.7	(56.9–66.4)	59.9	(55.7–63.9)	59.6	(55.2–63.8)	−2.1	−3.5	0.507
Florida	62.2	(56.5–67.5)	63.3	(60.3–66.2)	59.2	(55.1–63.1)	−3.0	−4.8	0.385
Georgia	65.9	(61.9–69.6)	66.3	(63.2–69.2)	70.2	(62.4–77.0)	4.3	6.5	0.309
Hawaii	60.4	(55.5–65.1)	60.7	(56.1–65.0)	64.0	(59.8–68.0)	3.6	5.9	0.266
Idaho	53.5	(49.4–57.5)	58.3	(52.8–63.6)	56.2	(51.4–60.8)	2.7	5.0	0.398
Illinois	62.0	(57.5–66.3)	64.0	(59.3–68.4)	65.0	(59.5–70.1)	3.0	4.8	0.401
Indiana	64.3	(60.7–67.8)	66.7	(61.9–71.2)	63.9	(60.3–67.3)	−0.4	−0.7	0.857
Iowa	57.6	(53.3–61.9)	62.1	(58.0–65.9)	66.1	(61.0–70.9)	8.5	14.7	0.012
Kansas	64.1	(60.7–67.3)	61.8	(57.6–65.9)	64.7	(62.1–67.3)	0.6	1.0	0.766
Kentucky	73.4	(68.9–77.5)	73.2	(67.7–78.2)	65.7	(61.4–69.7)	−7.7	−10.6	0.011
Louisiana	73.4	(68.3–78.0)	76.3	(72.2–79.9)	71.4	(67.4–75.0)	−2.0	−2.8	0.514
Maine	61.4	(56.0–66.5)	58.9	(55.6–62.0)	59.8	(56.2–63.3)	−1.6	−2.6	0.623
Maryland	66.7	(60.3–70.3)	64.3	(61.0–67.4)	67.4	(64.1–70.4)	0.7	1.0	0.796
Massachusetts	58.1	(54.2–62.0)	61.7	(59.1–64.2)	59.3	(55.9–62.6)	1.2	2.0	0.655
Michigan	60.8	(58.1–63.4)	62.8	(59.4–66.0)	65.6	(62.0–69.0)	4.8	7.9	0.032
Minnesota	65.6	(56.0–74.0)	66.2	(59.5–72.3)	72.7	(66.0–78.5)	7.1	10.9	0.207
Mississippi	70.3	(64.5–75.4)	73.1	(69.5–76.4)	72.4	(68.2–76.2)	2.1	3.0	0.545
Missouri	65.7	(60.8–70.2)	61.3	(57.5–64.9)	63.3	(58.5–67.8)	−2.4	−3.6	0.482
Montana	52.0	(48.0–55.9)	56.5	(52.6–60.4)	58.4	(52.3–64.3)	6.4	12.4	0.08
Nebraska	65.6	(60.9–70.1)	62.4	(57.8–66.8)	58.9	(55.3–62.4)	−6.7	−10.3	0.023
Nevada	49.9	(44.7–55.1)	52.4	(47.5–57.3)	55.3	(49.4–61.1)	5.4	10.8	0.178
New Hampshire	59.6	(55.9–63.1)	57.2	(53.5–60.9)	57.6	(53.3–61.8)	−2.0	−3.3	0.495
New Jersey	60.9	(58.1–63.6)	62.0	(58.4–65.5)	64.7	(60.2–69.0)	3.8	6.3	0.147
New Mexico	60.0	(53.6–66.0)	61.9	(56.8–66.6)	55.8	(52.0–59.5)	−4.2	−7.0	0.261
New York	59.7	(56.2–63.0)	61.0	(57.0–64.8)	61.2	(57.3–65.0)	1.5	2.6	0.546
North Carolina	63.5	(61.5–65.5)	67.5	(64.2–70.5)	66.7	(62.8–70.4)	3.2	5.0	0.152
North Dakota	63.5	(58.8–68.0)	57.6	(53.8–61.4)	64.6	(59.8–69.1)	1.1	1.7	0.75
Ohio	63.3	(59.2–67.3)	63.4	(60.4–66.2)	65.7	(60.5–70.6)	2.4	3.7	0.474
Oklahoma	67.2	(63.3–70.8)	64.3	(60.4–68.0)	63.8	(59.8–67.6)	−3.4	−5.1	0.218
Oregon	56.8	(53.4–60.1)	56.1	(51.2–61.0)	58.1	(53.0–63.1)	1.3	2.4	0.667
Pennsylvania	62.2	(59.1–65.3)	64.2	(60.5–67.8)	64.3	(60.4–68.1)	2.1	3.3	0.412
Rhode Island	63.9	(60.0–67.6)	63.5	(58.6–68.1)	58.1	(53.8–62.2)	−5.8	−9.1	0.045
South Carolina	69.3	(65.7–72.7)	69.3	(65.5–72.8)	65.7	(61.9–69.3)	−3.6	−5.2	0.163
South Dakota	60.2	(56.4–63.9)	60.0	(56.3–63.6)	57.8	(53.1–62.3)	−2.4	−4.0	0.429
Tennessee	75.5	(68.5–81.4)	72.3	(66.8–77.1)	74.1	(66.8–80.3)	−1.4	−1.9	0.769
Texas	60.3	(56.2–64.3)	64.7	(61.9–67.3)	61.5	(58.2–64.7)	1.2	2.0	0.641
Utah	56.2	(51.4–60.8)	54.0	(48.9–59.0)	54.5	(51.2–57.7)	−1.7	−3.0	0.563
Vermont	57.6	(54.2–60.9)	62.3	(56.0–68.2)	55.6	(51.7–59.4)	−2.0	−3.5	0.445
Virginia	64.2	(59.2–69.0)	64.5	(59.5–69.2)	64.9	(59.6–69.9)	0.7	1.1	0.844
Washington	55.7	(53.5–57.8)	56.3	(54.3–58.3)	55.4	(52.8–57.9)	−0.3	−0.5	0.875
West Virginia	65.6	(62.0–69.1)	72.2	(67.4–76.5)	70.5	(66.0–74.5)	4.9	7.4	0.089
Wisconsin	62.1	(57.3–66.7)	59.1	(54.9–63.2)	59.4	(54.8–63.8)	−2.7	−4.4	0.416
Wyoming	60.2	(55.2–64.9)	59.9	(55.0–64.7)	55.0	(51.6–58.4)	−5.2	−8.6	0.087

**Abbreviation:** CI = confidence interval.

* In this report, persons identified as Hispanic might be of any race. Persons identified as black, white, Asian/Pacific Islander, or American Indian/Alaska Native are non-Hispanic. The five racial/ethnic categories are mutually exclusive.

## Footnotes

* Details on BRFSS methodology, sampling procedures, design, and quality are available at http:/www.cdc.gov/brfss.

† In this report, persons identified as Hispanic might be of any race. Persons identified as black, white, Asian/Pacific Islander, or American Indian/Alaska Native are non-Hispanic. The five racial/ethnic categories are mutually exclusive.

§ Available at http://www.cdc.gov/dhdsp/programs/nhdsp_program/goals.htm.

¶ Available at http://www.thecommunityguide.org/cvd/teambasedcare.html.

** Information available at http://www.cdc.gov/dhdsp/programs/nhdsp_program/index.htm.
